# A missense mutation in the *agouti signaling protein* gene *(ASIP)* is associated with the no light points coat phenotype in donkeys

**DOI:** 10.1186/s12711-015-0112-x

**Published:** 2015-04-08

**Authors:** Marie Abitbol, Romain Legrand, Laurent Tiret

**Affiliations:** Inra, Unité de Génétique Fonctionnelle et Médicale, Ecole nationale vétérinaire d’Alfort, Maisons-Alfort, 94700 France; Inserm, U955 IMRB, Equipe 10, Université Paris-Est, Créteil, 94000 France

## Abstract

**Background:**

Seven donkey breeds are recognized by the French studbook and are characterized by a black, bay or grey coat colour including light cream-to-white points (LP). Occasionally, Normand bay donkeys give birth to dark foals that lack LP and display the no light points (NLP) pattern. This pattern is more frequent and officially recognized in American miniature donkeys. The LP (or pangare) phenotype resembles that of the light bellied agouti pattern in mouse, while the NLP pattern resembles that of the mammalian recessive black phenotype; both phenotypes are associated with the *agouti signaling protein* gene (*ASIP*).

**Findings:**

We used a panel of 127 donkeys to identify a recessive missense c.349 T > C variant in *ASIP* that was shown to be in complete association with the NLP phenotype. This variant results in a cysteine to arginine substitution at position 117 in the ASIP protein. This cysteine is highly-conserved among vertebrate ASIP proteins and was previously shown by mutagenesis experiments to lie within a functional site. Altogether, our results strongly support that the identified mutation is causative of the NLP phenotype.

**Conclusions:**

Thus, we propose to name the c.[349 T > C] allele in donkeys, the *a*^*nlp*^ allele, which enlarges the panel of coat colour alleles in donkeys and *ASIP* recessive loss-of-function alleles in animals.

**Electronic supplementary material:**

The online version of this article (doi:10.1186/s12711-015-0112-x) contains supplementary material, which is available to authorized users.

## Background

**Figure 1:**
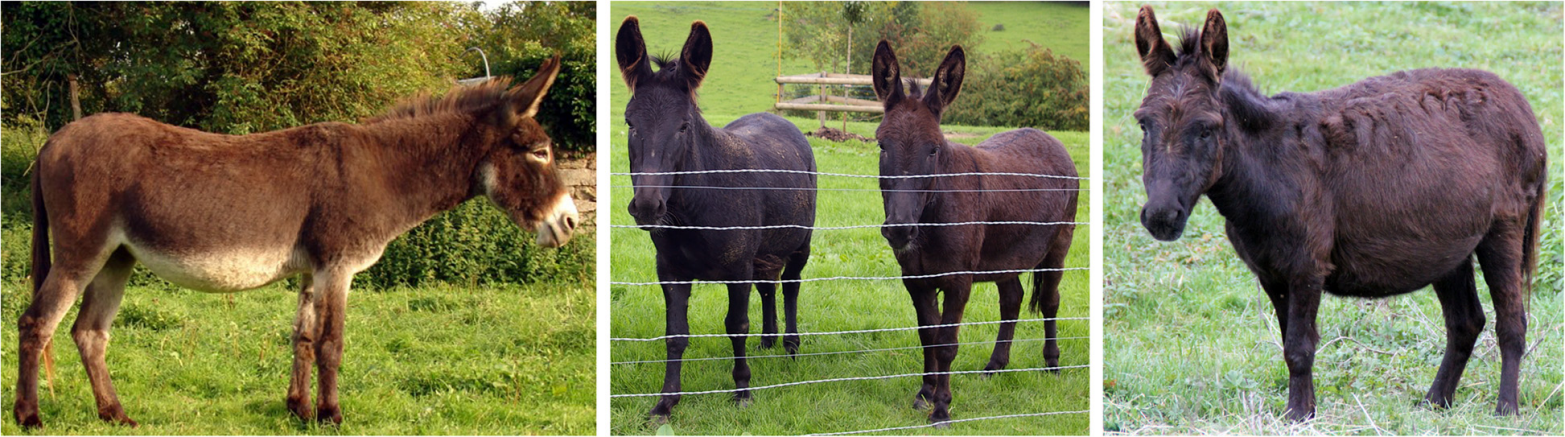
**No light points phenotype in donkeys.** Most coloured donkeys show a light cream to white coat on their belly and around their eyes and muzzle (Normand donkey, left). Bay Normand donkeys occasionally give birth to no light points (NLP) donkeys that are not officially recognized by the studbook (NLP donkeys, middle). The NLP phenotype is recognized in American miniature donkeys (NLP American miniature donkey, right).

Mutations in the gene *ASIP* (*agouti signaling protein*) result in various coat patterns in domestic mammals (http://omia.angis.org.au) including mouse (www.informatics.jax.org), dog [1], cat [2], rabbit [3], horse [4], sheep [5-8], rat [9] and alpaca [10]. Only a few coat colours, patterns and textures have been described in domestic donkeys (*Equus asinus*). In donkeys, the coat colour can be white or coloured, i.e. black, bay, grey and red with or without white spotting; hair texture is variable and includes the longhair phenotype, in addition to the common shorthair phenotype. Recently, we started to investigate the molecular aetiology of these phenotypes and identified three underlying loss-of-function mutations in the *MC1R* (*melanocortin 1 receptor*) and *FGF5* (*fibroblast growth factor*) genes that are responsible respectively, for the red colour and longhair phenotype in donkeys [11,12]. Most coloured donkeys are born with a pangare or light points (LP) pattern that associates cream to grey-white hair on the belly, around the muzzle and around the eyes. In the American miniature donkey breed, all coat colours and patterns are admissible and foals with a no light points (NLP) coat are often obtained from LP breeding stock (Figure 1). This has led breeders to suspect a recessive inheritance pattern for the NLP pattern. For the seven French donkey breeds (Pyrenean, Berry Black, Poitou, Cotentin, Provence, Bourbonnais and Normand), the NLP pattern is not officially recognized. However, dark NLP donkeys are occasionally born to bay Normand parents (Figure 1).

## Methods

### Animals and ethics statement

One hundred and twenty seven donkeys from six breeds were included in the study. They were all sampled in France between September 2012 and October 2014 and included Normand (n = 35), Provence (n = 14), Poitou (n = 13), Pyrenean (n = 13) and Berry Black (n = 2) breeds and miniature donkeys (n = 50).

All donkeys were included at the owners’ request. Pictures and hair samples were sent directly by owners or collected by a veterinarian (MA). All animals were client-owned donkeys on which no harmful invasive procedure was performed; thus, according to the legal definitions in Europe (Subject 5f of Article 1, Chapter I of the Directive 2010/63/UE of the European Parliament and of the Council), no animal experiment was carried out.

### DNA extraction

DNA was extracted from hair roots using a Maxwell® 16 Instrument (Promega Corporation, Madison, USA), according to the manufacturer’s protocol.

### Sequencing of *ASIP* and genotyping

Reference genomic sequences were extracted from Ensembl (Equine *ASIP* gene, ENSECAG00000004241). PCR and sequencing primers were designed using Primer3 [13]. The three exons were amplified using three sets of primers [See Additional file 1: Table S1]. PCR amplicons were sequenced using Sanger dideoxy sequencing in both forward and reverse directions by GATC Biotech (GATC Biotech AG, Konstanz, Germany). Electropherograms were manually inspected with Chromas Lite (Technelysium Pty Ltd, South Brisbane, Australia). Multiple alignments were performed using Multalin ([14]; http://multalin.toulouse.inra.fr).

### Protein sequence comparisons and impact of sequence variations

ASIP amino acid sequences from various species were collected from Ensembl (mouse: ENSMUST00000109697; horse: ENSECAT00000004772; cow: ENSBTAT00000048322; sheep: ENSOART00000010128; dog: ENSCAFT00000038625; cat: ENSFCAT00000011040; human: ENST00000568305; chicken: ENSGALT00000044768; zebrafish: ENSDART00000113083). Multiple alignments were performed using Multalin ([14]; http://multalin.toulouse.inra.fr). The putative impact of missense mutations was assessed using three different software, namely PolyPhen-2 (HumVar-trained PolyPhen-2 designed to distinguish mutations with drastic effects from other variations, including abundant mildly deleterious alleles, ([15]; http://genetics.bwh.harvard.edu/pph2/), SNAP ([16]; www.rostlab.org/services/snap/submit) and PROVEAN ([17]; http://provean.jcvi.org/seq_submit.php). The different domains of ASIP were schematized according to the previously published ASIP structure in mouse [18].

### Accession numbers

Genomic coding sequences of *ASIP* from bay LP and NLP Normand donkeys were submitted to GeneBank. Accession numbers are KJ126712 for the LP allele and KP717040 for the NLP mutant allele.

## Findings

Because whole-genome mapping tools are still lacking for donkey, we decided to screen directly for variants that affect ASIP function in two NLP and two LP control Normand donkeys that originated from a comprehensive panel of 127 donkeys from six breeds. The Ensembl *ASIP* equine genomic sequence was used to design three sets of intronic primers [See Additional file 1: Table S1] that allowed successful amplification of the three exons of the donkey *ASIP* gene. Then, we sequenced the three *ASIP* exonic amplicons and performed pair-wise base-to-base comparisons of the sequences between the LP and NLP donkeys and a horse reference sequence; we found that the coding sequences and the sequences that covered intron-exon boundaries were highly conserved between horse and donkey and detected only two variants between donkey sequences and the bay horse reference sequence [See Additional file 1: Table S2]. Only the c.349 T > C SNP (single nucleotide polymorphism) produced a substitution p.(Cys117Arg) and was consistent with the recessive mode of inheritance of the NLP pattern. Indeed both NLP donkeys were homozygous *C/C* for the mutant allele of c.349 T > C SNP, while one control donkey was heterozygous *C/T*, and the other control donkey and the bay horse were homozygous *T/T* for the reference allele [See Additional file 1: Table S2]. The second non-coding variant was located in the 3’UTR (untranslated region) region of the *ASIP* gene and was not associated with the NPL phenotype [See Additional file 1: Table S2].

PROVEAN, PolyPhen-2 and SNAP predicted that the p.(Cys117Arg) substitution was deleterious. Hence, the complete cohort of 127 donkeys was genotyped for SNP c.349 T > C. The nine NLP donkeys, including three NLP Normand and six NLP miniature donkeys, were all homozygous *C/C*, while the 118 LP donkeys were either homozygous *T/T* (n = 104) or heterozygous *C/T* (n = 14). The three NLP Normand donkeys were born to a single male mated with three females, which were all four heterozygous *C/T*. The complete concordance between the recessively-inherited NLP pattern and the c.349 T > C variant (Table 1) supported our hypothesis that this SNP is associated with the NLP trait (Chi square test p = 1.86 × 10^−29^).

**Table 1:** **Genotypes for the c.349T > C variant in donkeys**

	***T/T***	***T/C***	***C/C***	**Total**
LP Berry Black donkeys	2	0	0	2
LP Pyrenean donkeys	13	0	0	13
LP Poitou donkeys	13	0	0	13
LP Provence donkeys	12	2	0	14
LP Normand donkeys	26	6	0	32
NLP Normand donkeys	0	0	**3**	3
NLP miniature donkeys	0	0	**6**	6
LP miniature donkeys	38	6	0	44
Total	104	14	**9**	127

LP: light points, NLP: no light points phenotypes. Homozygous mutants are bolded.

**Figure 2:**
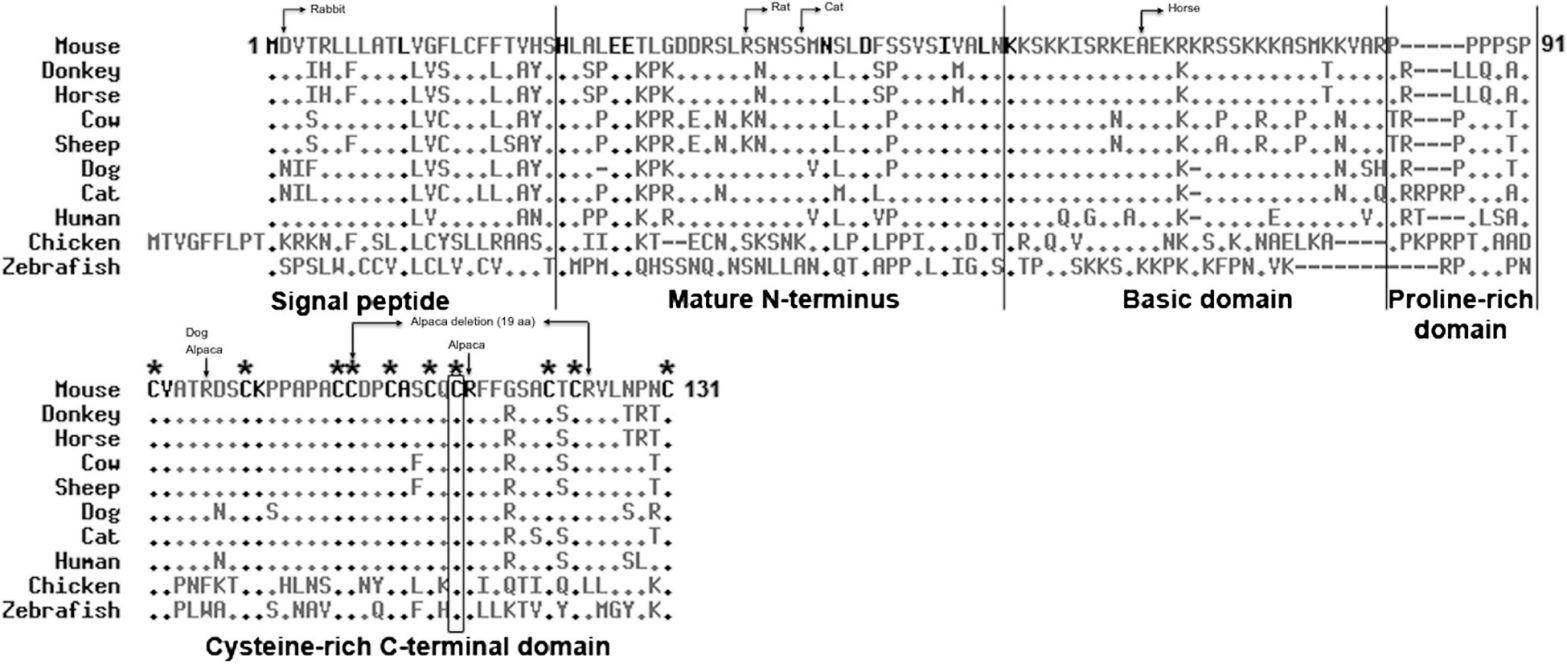
**Alignment between ASIP protein sequences from 10 vertebrate species.** ASIP amino acid sequences from various species were collected from Ensembl (mouse: ENSMUST00000109697; horse: ENSECAT00000004772; cow: ENSBTAT00000048322; sheep: ENSOART00000010128; dog: ENSCAFT00000038625; cat: ENSFCAT00000011040; human: ENST00000568305; chicken: ENSGALT00000044768; zebrafish: ENSDART00000113083). ASIP sequences are identified with the name of the species on the left. The mouse ASIP sequence (reference sequence) is at the top of the alignment. Non-conserved residues in the 10 species analyzed are shown in grey. Conserved residues are indicated in black within the reference sequence and represented by black dots in other sequences. Dashes represent deletions. The conserved C117 residue in donkey ASIP that corresponds to the murine C113 residue is circled. The 10 functional cysteine residues that have been shown to be involved in the activity of ASIP are indicated with stars. *Non-agouti* mutations identified in other domestic mammalian species are reported above the corresponding position. Dog and alpaca mutations consist in missense mutations and a 19 amino acids (aa) in-frame deletion. Rabbit, rat, cat and horse mutations are frameshifts. Mouse and sheep regulatory mutations are not shown.

To estimate the functional importance of the donkey ASIP cysteine 117 amino acid, we aligned the donkey ASIP protein sequence with the ASIP sequences of nine vertebrates and found that is was fully conserved (Figure 2). This result confirmed the 100% conservation previously reported for the 10 cysteine amino acids of the C-terminal Cys-rich domain of ASIP [18-21] the functional role of which was investigated by mutagenesis experiments. Perry and collaborators reported that in mouse, 13 mutated ASIP proteins displayed a partial (n = 4) or a total (n = 9) loss of activity [21]. In particular, they found that eight of the 10 cysteines located in the Cys-rich C-terminal tail of ASIP, including the murine cysteine 113 that corresponds to the donkey cysteine 117, were critical for protein activity [21]. Altogether these results strongly support that, in donkeys, the ASIP cysteine 117 has an essential role for ASIP function.

In conclusion, the complete association between the c.349 T > C mutation and the NLP phenotype and its inheritance pattern, on the one hand, and the high probability that the resulting substitution of the conserved cysteine 117 residue leads to loss of function of the mutated protein, on the other hand, support that this mutation is responsible for the NLP phenotype in donkeys. We thus propose to name the c.[349 T > C] allele, which can be easily detected with a DNA test, the *a*^*nlp*^ allele in donkeys.

## Additional file

Additional file 1: Table S1. PCR and sequencing primers. Sequences and PCR temperatures from the intronic primers that were used to amplify and sequence the three *ASIP* coding exons. **Table S2.** Genomic variants in *ASIP* identified between donkey sequences and the horse reference sequence. Sequence variants in *ASIP* identified between two NLP donkeys, two control donkeys and the horse reference sequence (coding sequences and 5’end of the 3’UTR).
