# Supplementary material for: A missense mutation in the agouti signaling protein gene (ASIP) is associated with the no light points coat phenotype in donkeys
Source: Genet Sel Evol. 2015 Apr 8;47(1):28. doi: 10.1186/s12711-015-0112-x (PMC4389795; doi:10.1186/s12711-015-0112-x)
Supplement: Additional file 1: Table S1. — PCR and sequencing primers. Sequences and PCR temperatures from the intronic primers that were used to amplify and sequence the three ASIP coding exons. Table S2. Genomic variants in ASIP identified between donkey sequences and the horse reference sequence. Sequence variants in ASIP identified between two NLP donkeys, two control donkeys and the horse reference sequence (coding sequences and 5’end of the 3’UTR). [file 12711_2015_112_MOESM1_ESM.docx]

**Additional file 1**

**Table S1. PCR and sequencing primers**

| Exon | Primers | Primer sequence | Tm | PCR product |
| --- | --- | --- | --- | --- |
| *ASIP*exon1 | *ASIP*exon1F | acctgcctgactgccttc | 60°C | 253bp |
|  | *ASIP*exon1R | ccttctcatgggctggag |  |  |
| *ASIP*exon2 | *ASIP*exon2F | tgagtcccctccactcct | 60°C | 202bp |
|  | *ASIP*exon2R | tcctagccagagcttgagg |  |  |
| *ASIP*exon3 | *ASIP*exon3F | cggcctccatagtccaaag | 60°C | 419bp |
|  | *ASIP*exon3R | cccgcctcctagaagctc |  |  |

F: forward. R: reverse. Tm: melting temperature. bp: base pairs.

**Table S2. Genomic variants in *ASIP* identified between donkey sequences and the horse reference sequence**

|  | c.349T>C  exon 3 | c.*4C>A  3’UTR |
| --- | --- | --- |
| Ensembl equine sequence | T | C |
| NLP Normand 1 | *C/C* | *C/C* |
| NLP Normand 2 | *C/C* | *C/C* |
| Bay Normand control 1 | *T/C* | *C/C* |
| Bay Normand control 2 | *T/T* | *C/A* |
| Consequence | p.(Cys117Arg) | p.(=) |
| Prediction | Damaging# |  |

NLP: no-light-points; #: Polyphen-2, SNAP and PROVEAN predictions.
